# Mutation in *cpsf6/CFIm68* (*Cleavage and Polyadenylation Specificity Factor Subunit 6*) causes short 3'UTRs and disturbs gene expression in developing embryos, as revealed by an analysis of primordial germ cell migration using the medaka mutant *naruto*

**DOI:** 10.1371/journal.pone.0172467

**Published:** 2017-03-02

**Authors:** Takao Sasado, Hisato Kondoh, Makoto Furutani-Seiki, Kiyoshi Naruse

**Affiliations:** 1 Laboratory of Bioresources, National Institute for Basic Biology, Aichi, Japan; 2 Faculty of Life Sciences, Kyoto Sangyo University, Kyoto, Japan; 3 School of Medicine, Yamaguchi University, Ube, Japan; Rutgers New Jersey Medical School, UNITED STATES

## Abstract

Our previous studies analyzing medaka mutants defective in primordial germ cell (PGC) migration identified *cxcr4b* and *cxcr7*, which are both receptors of the chemokine *sdf1/cxcl12*, as key regulators of PGC migration. Among PGC migration mutants, *naruto* (*nar*) is unique in that the mutant phenotype includes gross morphological abnormalities of embryos, suggesting that the mutation affects a broader range of processes. A fine genetic linkage mapping and genome sequencing showed the *nar* gene encodes Cleavage and Polyadenylation Specificity Factor subunit 6 (CPSF6/CFIm68). CPSF6 is a component of the Cleavage Factor Im complex (CFIm) which plays a key role in pre-mRNA 3'-cleavage and polyadenylation. 3'RACE of *sdf1a/b* and *cxcr7* transcripts in the mutant embryos indicated shorter 3’UTRs with poly A additions occurring at more upstream positions than wild-type embryos, suggesting CPSF6 functions to prevent premature 3’UTR cleavage. In addition, expression of the coding region sequences of *sdf1a/b* in *nar* mutants was more anteriorly extended in somites than wild-type embryos, accounting for the abnormally extended distribution of PGCs in *nar* mutants. An expected consequence of shortening 3'UTR is the escape from the degradation mechanism mediated by microRNAs interacting with distal 3’UTR sequence. The abnormal expression pattern of *sdf1a* coding sequence may be at least partially accounted for by this mechanism. Given the pleiotropic effects of *nar* mutation, further analysis using the *nar* mutant will reveal processes in which CPSF6 plays essential regulatory roles in poly A site selection and involvement of 3'UTRs in posttranscriptional gene regulation in various genes *in vivo*.

## Introduction

Primordial germ cells (PGCs), the precursors of sperm and egg, play central roles in inheritance in sexually reproducing organisms. In many animals, PGCs originate in embryonic tissue remote from where the gonads will form early in development and thereafter migrate over a long distance while interacting with somatic tissues, eventually colonizing in the gonadal primordium. Though the migratory routes of PGCs exhibit animal species-dependent variations, common molecular mechanisms regulating PGC migration have been recently identified in vertebrates [1–3]. Recently, research performed on small teleost fish, predominantly utilizing medaka (*Oryzias latipes*) and zebrafish (*Danio rerio*), has elucidated that a chemokine, CXCL12/SDF1 (C-X-C motif chemokine ligand 12/Stromal cell-derived factor 1), which works as a chemoattractant for migrating PGCs, plays important roles in the guidance of PGC migration [4–6]. Several paralogues of the chemokine and its receptors exist in teleost fish; their respective roles in PGC migration are explained in brief below. SDF1 is expressed in the somatic tissues of the migratory route of the PGCs in developing embryos. PGCs expressing the receptor CXCR4 (C-X-C motif chemokine receptor 4) on their plasma membrane migrate in response to the SDF1 gradient. The proper gradient of SDF1 in the somatic environment is thought to be formed by another somatically expressed SDF1-receptor, CXCR7, promoting the internalization and degradation of SDF1 as a sequestration receptor [7]. miRNA-mediated post-transcriptional gene regulation of *sdf1* and *cxcr7* has also been indicated as facilitating proper gradient formation of SDF1 [8–10]. Research has shown that this SDF1-CXCR4 chemokine signaling plays an important role in the regulation of migration and colonization of PGCs to the gonads not only in fish but also in a wide variety of vertebrates such as mice [11], chickens [12] and frogs [13].

Thus, it is apparent that CXCR4-dependent SDF1 signaling has a critical role in the guidance of PGCs to the developing gonads in vertebrates; however, this may not be the only signaling system involved in directing the migration of PGCs. In recent years, research has revealed there is more complicated crosstalk between the chemokine interactions than was previously thought. That is, the two receptors of SDF1, CXCR4 and CXCR7 individually interact not only with SDF1 but also several other ligands including chemokines; moreover the ligands interact with other chemokine receptors affecting tumor cell growth and metastasis, as well as regulation of chemotaxis in the immune cells and hematopoietic progenitor cells [14–16]. For example, another ligand for CXCR7, CXCL11 also interacts with CXCR3 [17], and one of several ligands for CXCR4, the cytokine macrophage migration inhibitory factor (MIF) also interacts with CXCR2 [18]. In addition, CXCL14 binds to CXCR4 and inhibits CXCL12-CXCR4 signaling [19] and CXCL12 signaling mediated by CXCR4 regulates and plays an important role not only in PGC migration but also a wide range of biological phenomena and developmental processes in vertebrates; such as leukocyte chemotaxis, homing of hematopoietic cells, blood vessel formation, neuronal patterning, and tumor cell growth and metastasis [5]. Therefore, findings obtained from analyses of the molecular mechanisms involved in PGC migration and the concurrent interactions with surrounding somatic cells in the developing embryo will give new insights into the understanding of a wide range of biological phenomena in vertebrates involving directed cell migration relying on the same molecular mechanisms.

In order to elucidate the mechanisms underlying the development and migration of PGCs, we undertook the first large-scale and systematic mutant screening focusing on early-stage development of PGCs in vertebrates, using medaka. As a result of the screening, we identified a battery of mutations affecting the migration and development of the PGCs [20]. This systematic screening focusing on the specific phenotype in the early-developing stage was facilitated by the biological features of medaka as a model animal in vertebrates: such as its externally-fertilized transparent embryos, short generation time, easy husbandry and fecundity [21–25]. From the collected mutations, we analyzed two mutations with defective PGC migration and identified the responsible genes as *cxcr4b* and *cxcr7* respectively [26]. Combining our findings with those of other recent studies on medaka, it became apparent that *sdf1a*, *sdf1b* and *cxcr7* expressed in the somatic cells where the PGCs migrate and *cxcr4b* expressed in the PGCs were indispensable to the PGC-directed migration and gathering to the ventrolateral areas of the presumptive gonads [27–30].

In the present study, we focused on a recessive homozygous lethal mutation affecting PGC migration, named *naruto* (*nar*) identified in the previous mutagenesis screening [20]. The mutant was so named because of its excessively curved trunk, which is reminiscent of the renowned whirlpools streams of the Naruto Straight of Japan. Surprisingly, the gene responsible for the mutation is *cleavage and polyadenylation specificity factor subunit 6* (*cpsf6/CFIm68*), which is a component of the Cleavage Factor Im complex (CFIm) that plays a key role in pre-mRNA 3'-processing and is required for 3' RNA cleavage and polyadenylation processing. How then does this gene, thought to be used commonly and expressed ubiquitously, disturb the directed migration of the PGCs? This study shows that the *cpsf6* mutation results in the shortening of several mRNAs in the 3' UTRs, including the chemokines, *sdf1a*, *sdf1b* and *cxcr7* in developing embryos. Furthermore, the tissue areas expressing *sdf1a* and *sdf1b* are abnormally extended more towards the anterior area in the mutant somite in PGC migration stage embryos.

We propose the following model to link the mutated gene’s function and the mutant phenotype. mRNA 3'UTR recognized by microRNAs (miRNAs) is involved in the regulation of mRNA stability and translational control [31–33]. It is possible that the shortened 3'UTR of *sdf1a/b* allows its mRNAs to escape from the miRNA-mediated transcript degradation in the anterior mesodermal tissues. This abnormally sustained *sdf1a/b* mRNAs in the anterior mesodermal tissues in *nar* mutant embryos will perturb the generation of the SDF1 gradient along the embryo axis, which is required for the posterior migration of the PGCs. Other sets of genes will also be affected in *nar* mutants, which are causative for additional phenotypes, but we will confine our present study to the effect on PGC migration. In any event, this is the first study to show dysfunction of one of the component CFIm, CPSF6, affects poly A site selection and disturbs gene expression in the developing vertebrate embryos.

## Materials and methods

### Fish

The medaka fish (*Oryzias latipes*) used in this research were maintained as described previously [21], and their developmental stages determined according to the stage table [34]. The Cab line and the Cab-derived *nar* mutant were as previously described [20, 35]. All medaka strains used in this experiment are available from the National Bioresource Project Medaka [36]. Animal experiments were approved by the Institutional Animal Care and Use Committee of the National Institutes of Natural Sciences and followed the guidelines of the National Institute for Basic Biology; approval numbers were 12A085, 13A158, and 14A166.

### Genetic linkage analysis

**Fig 1:**
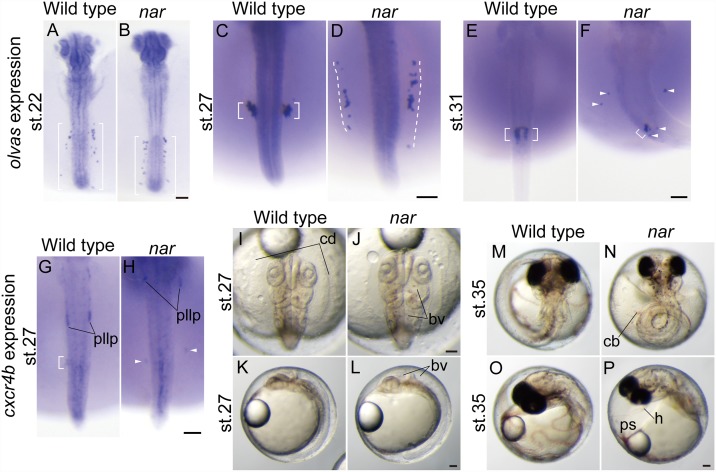
Characterization of the *naruto*^*j113-2B*^ (*nar*^*j113-2B*^) mutation in medaka. (A-F) Altered PGC distributions in the *nar* mutants. PGCs were labeled by *in situ* hybridization with a medaka *vasa* (*olvas*) probe. At st. 22, PGCs are aligned bilaterally in the posterior trunk region in the *nar* mutant embryo (B, brackets) similar to the wild type (A, brackets). In the wild-type embryo at st.27, PGCs are clustered at the ventrolateral region of the prospective gonad (C, brackets), in contrast to the PGCs which are scattered anterior and to the lateral sides of the normal clustering position on the yolk sac (D, underlined by dashed lines) of the *nar* mutant. PGCs are gathered in the medial areas in the wild-type embryos at st. 31 (E, brackets). In the *nar* mutant embryos at the same stage (F), a part of the PGCs are gathered in the normal position (brackets), whereas some PGCs are scattered in the ectopic area (arrowheads). (G-H) Whole mount *in situ* hybridization of *cxcr4b* in wild-type (G) and *nar* (H) embryos at st. 27. The *cxcr4b* transcript distribution is observed in posterior lateral line primordia (pllp). The posterior lateral line primordia are distributed remarkably anterior in the *nar* mutant embryo (H) compared to the wild-type embryo (G). Weak *cxcr4b* transcript distribution is also seen in PGCs in the wild type (G, bracket) and is more laterally positioned in the *nar* mutant embryo (H, arrowheads). (I-P) Pictures of the live embryos showing the *nar* morphogenetic defects and their normal siblings. The *nar* mutant embryos exhibit enlarged brain ventricles (bv) at st.27 (I-L), a curved trunk, an enlarged pericardial space (ps) and a thin heart (h) at st. 35 (M-P). After st. 27 (N), blood circulation is stopped and clogged blood (cb) is observed. Dorsal views are shown in the orientation of anterior toward the top in the A-J, M-N. Lateral views are shown in the orientation of anterior toward the top in the K-L, O-P. Flat-mount embryos at st. 22 are shown in A-B. Scale bars indicate 100 μm.

The genetic linkage analysis between polymorphic markers and the *nar*^*j113-2B*^ mutation was done using the same technique as in our previous report [26]. The F2 progeny of the mapping cross were sorted by observing the morphology at st.27 (Fig 1I–1L). Proteinase K digested crude extracts were used for genomic DNA templates for PCR [37]. The embryos were fixed and stored in methanol at -20°C, dried by heating at 70°C for 15min, digested in 25μl 2mg/mL proteinase K in TE (10mM Tris-HCl, pH8.0, 1mM EDTA) at 55°C for 3hrs, heated at 95°C for 10min to deactivate the proteinase K, and mixed with 75μl sterilized distilled water. The supernatants of the crude extracts were used as the PCR templates at 1/10 volume of the final PCR reaction mixture. Primers for the polymorphic markers were designed according to Kimura et al. (2010) [38]. The primer information is noted in the supplemental information (S1 Table). PCR products were separated using an automated microchip electrophoresis-system (MultiNA MCE-202, Shimadzu, Kyoto, Japan) in the genotyping of the F2 panel.

### Genomic DNA sequence determination

Full-length cDNA clones of medaka *cpsf6* provided from NBRP Medaka [36, 39], *oleb23l11* and *oleb36o05* with 587b and 2004b 3'UTR respectively, were sequenced (GenBank LC147085, LC148409) and 10 exons with 1656bp Open Reading Frames (ORF) spanning exon 1~9 were determined. Two genomic sequence sets corresponding to the exon 1~9, including splicing acceptor and donor sites, were amplified from the genomic DNA of 10 embryos taken from *nar* mutants and also 10 embryos of the Cab wild type, using Ex-Taq polymerase (Takara, Kyoto, Japan) and the DNA sequences of the PCR amplified fragments were determined according to Takehana et al. (2005) [40]. The primers used in the amplification of exon 2 with the *nar*^*j113-2B*^ mutation in the splice donor site were 5'- TGCATTTCTACATCCCCAGTT and 5' TGAAGCTAAAGCCTCGGTGT.

### Genotyping for *nar* mutation

**Fig 2:**
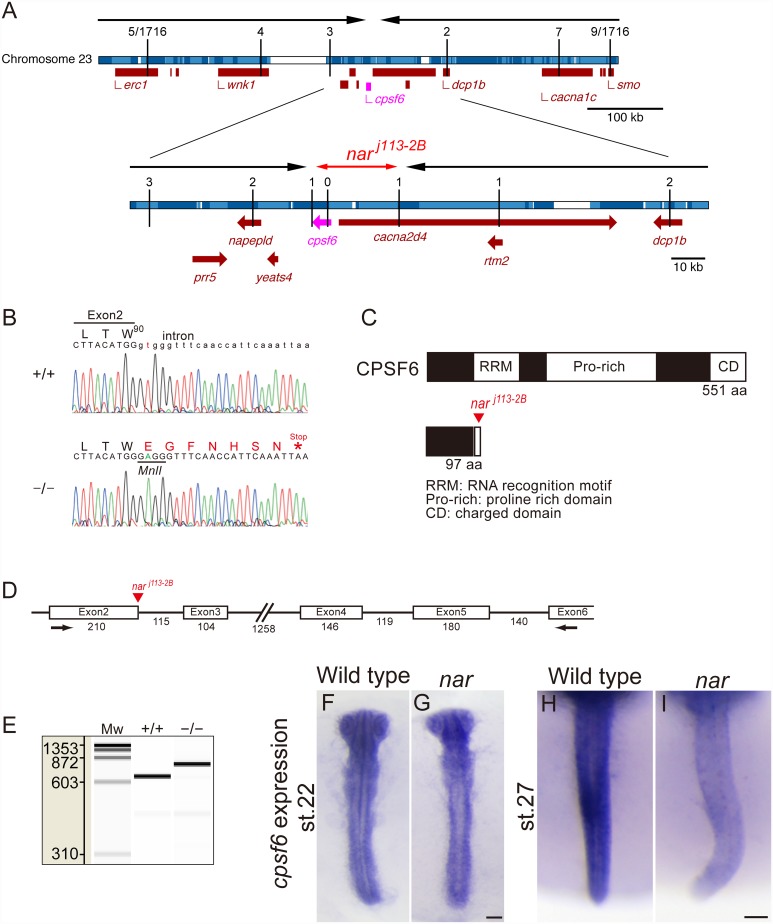
Identification of the *nar*^*j113-2B*^ mutation in a *cpsf6* gene. (A) Genetic linkages between polymorphic markers on chromosome 23 and the *nar*^*j113-2B*^ mutation. Recombination frequencies at the markers are indicated on the top of the vertical solid lines indicating each marker position on the chromosome 23 (horizontal bar). The *nar*^*j113-2B*^ was mapped between the *erc1* and the *smo* loci on chromosome 23 using a 1716 recombination density F2 panel. Further fine mapping using 5 or 9 recombinants at the *erc1* or the *smo* loci respectively resulted in narrowing down (horizontal black arrows) the *nar* candidate locus to within the 33kb region (double-headed horizontal red-arrow) containing the *cpsf6*. The chromosome information with annotated genes is referred from the Ensembl *Oryzias latipes* release version 63.1 [80, 81]. The individual contigs that make up the genomic assembly are colored in light or dark blue, and the gaps on the genome sequence are colored in white. The arrows of the annotated genes on the genome indicate the direction of the genes. (B) A local genome-sequence data set comparing the wild type (+/+) and *nar*^*j113-2B*^ homozygotes (-/-) demonstrating the T to A mutation at a splice donor site of the exon 2 in the *cpsf6* in the *nar*^*j113-2B*^ mutants. This mutation creates a *MnlI* site and an additional seven amino acids with a stop codon (red letters in the amino acid codes) from W^90^ in the expected CPSF6 amino acid sequence. (C) Predicted wild-type CPSF6 (upper) is 551 amino-acid length, contains highly conserved domains, RNA recognition motif (RPM), proline rich domain (Pro-rich) and charged domain (CD). Whereas, the predicted CPSF6 in the *nar* mutant embryos is 97 amino-acid length lacking almost all conserved domains (lower). (D) Genomic DNA structure of medaka *cpsf6* exon2-6. The numbers under the exons (rectangles) and introns (solid lines) indicate the DNA length (bp) in each region. (E) Electropherogram of RT-PCR amplified from total RNA of wild-type (+/+) or *nar* mutant (-/-) embryos at st.27 using primers indicated in D (arrows). This image was made by the automated microchip electrophoresis-system. The PCR product in the mutant is longer corresponding to the intron size between the exons 2 and 3 (115bp) than in the wild type. Mw: molecular weight marker, phiX174 DNA-*HaeIII* digest, with the sizes (bp) indicated on the left. (F-I) Whole-mount *in situ* hybridization using a *cpsf6* antisense probe corresponding to the open reading frame. At st. 22, *nar* mutants (F) have strong ubiquitous *cpsf6* transcript distribution in their whole body similar to wild type (G). The ubiquitous *cpsf6* transcript distribution continued in wild type at st. 27 (H), whereas it became weak in the mutant embryo (I). F, G: flat mounted embryos. Dorsal views are shown in the orientation of anterior toward the top. Scale bar, 100 μm.

The PCR fragments amplified from genomic DNA using primers, 5’- TCCAGTTCACGAGCAGATTG and 5’- CCTTTTGACTGGCCATTAGC were digested using *MnlI* to identify the *nar*^*j113-2B*^ mutation in *cpsf6* (Fig 2B). The 305bp or 225+80bp bands were observed in the products amplified from the wild-type and the *nar*^*j113-2B*^ alleles respectively after the restriction-enzyme digestion of the 418bp PCR products. For the genotyping of the live fish, 4mm^2^ pieces of tail-fin were cut under anesthesia achieved by submerging in 0.03% MS222 solution (Sigma, #A5040), digested by a 1/5 volume of proteinase K in prepared under the conditions mentioned in the "Genetic linkage analysis" paragraph, and the crude extracts were used for the PCR templates at 1/10 volume of the final PCR reaction mixture. For genotyping of the embryos stained by the whole mount *in situ* hybridization, st.27 embryos were treated using the same conditions mentioned in the "Genetic linkage analysis" paragraph, and embryos at st.22 were digested by a 1/5 volume of proteinase K prepared the same as previously mentioned.

### Injection of RNA

Capped RNAs were synthesized from the above mentioned full-length medaka *cpsf6* cDNA clones (*oleb23l11* and *oleb36o05*). The cDNA clones have artificial poly A sequences in the downstream of the 3'UTR [39]. The cDNA clones were linearized at *NotI* sites located just downstream of the poly A sequences, and were used as a template for *in vitro* transcription by T7 RNA polymerase using an mMessage mMachine kit (Ambion, Texas, USA). The full-length (1.7kb) *cpsf6* coding sequence was amplified from the *oleb23l11* cDNA clone using 5'-CGGGATCCACCATGGCGGACGGTGTGGAT and 5'-CGGAATTCCTAACGGTGGCGATATTCC primer set, digested with *BamHI* and *EcoRI*, and cloned in the *pCS2+* vector [41, 42] as mentioned in the previous paper [26]. The mRNA were synthesized same as above using the mMessage mMachine SP6 kit. The synthesized RNA was purified by RNeasy mini kit (Qiagen, Hilden, Germany), and injected into fertilized eggs as described previously [43] at 100 ng/μl.

### 3'RACE (Rapid Amplification of cDNA 3' Ends) and Reverse Transcription PCR (RT-PCR) analyses

Total RNAs from 50 embryos of wild type or the *nar* mutant at st.27 were extracted using Isogen (Nippon Gene, Toyama, Japan), digested with recombinant DNaseI (Takara) and purified by the RNeasy mini kit (Qiagen). The 3'RACE was carried out according to SMARTer RACE cDNA amplification kit instruction (Clontech, Palo Alto, CA, USA) with Superscript III first-strand synthesis system (Invitrogen) for the reverse transcription and Ex-Taq polymerase (Takara) for the PCR. Gene specific primers designed in the cording sequences for the 3'RACE were as follows, *sdf1a*: 5'-CATTCACACACCTAACTGCCCCTTCCA, *sdf1b*: 5'-AGCGCTCCATCAAAGAGCTCAAGTTCC, *cxcr7*: 5'-GGCACGTCTCATCGAGGCTCCTAACAT. To determine the DNA sequences of the 3' ends of the 3'RACE amplified cDNA products, the 3'RACE amplified cDNA products were reamplified by nested PCR using gene specific primers, *sdf1a*: 5'-GTGGCTTCAGCAGTACCTGA, *sdf1b*: 5'-AAGAACGCCATCAACAAGGT, *cxcr7*: 5'-ACCCGCCACTCATATCAAAG, separated by an agarose gel electrophoresis, and excised and purified using an Illustra GFX PCR DNA and gel band purification kit (GE Healthcare, Waukesha, WI, USA). The thermal cycling condition for the nested PCR was as follows: 94°C for 2 min; 20 cycles of 94°C for 30 sec, 68°C for 30 sec, and 72°C for 3 min; and 72°C for 5 min. The sequencing primers were as follows, *sdf1a*: 5'-ACCTAACCTCCTGCCGTCTT, 5'-GTAGCCGGCAAAAATCTGAG, *sdf1b*: 5'-CGTCGCTTTAACCTGCTTTC, 5'-AGCGGGAGATACAGGGAAGT, 5'-AACCCCAGGACAATGTGAAA, *cxcr7*: 5'-GGTCTGATGTCGTACCAGGAG, 5'-GCTTCAGTCCTGCTGTCTCA, 5'-CCAAAGCTCGACACCAAAAT.

The *cpsf6* transcripts in the wild-type and the *nar* mutant embryos at st.27 were amplified from the above mentioned cDNAs using primers designed in the exon2 and exon6 (Fig 2D), 5'-TCCAGTTCACGAGCAGATTG, 5'-ACGTGGAAGACCCTGCATT respectively, and were separated by the automated microchip electrophoresis-system (Fig 2E). The DNA sequences of each PCR amplified product were determined using the primer 5'-CCTTTTGACTGGCCATTAGC (data not shown).

### Whole mount *in situ* hybridization

*In situ* hybridization of whole-mount embryo specimens were performed as described previously [20]. Digoxigenin labeled antisense RNA probes to detect medaka *vasa* (*olvas*), *cxcr4b* orf and *cxcr7* orf were described previously [20, 26]. The probes for detecting orf or 3'UTR of medaka *cpsf6*, *sdf1a*, *sdf1b* and *cxcr7* were synthesized from cDNAs subcloned from full-length cDNA clones provided by NBRP Medaka. The full-length cDNA clones were as follows: *cpsf6*, *oleb36o05*; *sdf1a*, *olki24k03* (GenBank LC150876); *sdf1b*, *olbr34c09* (LC150602) and *cxcr7*, *olec12k21* (LC150747). The primers used for the subcloning of each clone were as follows: *cpsf6* orf, 5'-ATGGCGGACGGTGTGGAT, 5'-CTAACGGTGGCGATATTCC; *sdf1a* orf, 5'-ATGGATGTGAAGCTCTTTGC, 5'-TCAGATGATCTGCTTGGCTTTC; *sdf1a* distal 3'UTR, 5'-CAAAGCATTCTGCTCTGCTC, 5'-AAGCAGGATGGCTCAGTTGT; *sdf1b* orf, 5'-ATGGACGCCAAGCTGCTC, 5'-TTACATGTTCTGGCCATTGCG; *sdf1b* medial 3'UTR; 5'-GCAGACGAGTGACTGGAACC, 5'-CATCGAAAAAGTCACATTTGAAAG; *sdf1b* distal 3'UTR 5'-CCAGAATCAATGAGCAGCAG, 5'-TGTCAGAAGCTGCCTCAAAA and *cxcr7* distal 3'UTR, 5'-TTTCAGAAAAGCGTGTCAGC, 5'-CATCCGTGCATCAGAACAAT. The PCR fragments amplified using KOD-plus DNA polymerase (Toyobo, Osaka, Japan) were subcloned in pCRII-TOPO vector using TOPO TA cloning kit (Invitrogen, California, USA).

## Results

### *naruto*^*j113-2B*^ (*nar*^*j113-2B*^) mutation causes pleiotropic defects including in PGC migration and lateral line positioning

In wild-type embryos, the PGCs are first detected in the deep area throughout the animal pole at early gastrula stage (st.13) as fluorescence positive-cells in the EGFP-*nanos3* 3'UTR mRNA injection, which then move to the marginal zone of the embryonic shield at the mid-gastrula stage (st.15) [28]. Then the PGCs move to the posterior region of the embryonic body at the early-neurula stage (st.17) and subsequently form bilateral lines along the body axis in the posterior trunk region at st.22 (9 somite stage), as detected by *in situ* hybridization using an *olvas* (medaka *vasa* homolog) probe (Fig 1A, bracket). After this PGC lining-up stage, PGCs make a posterior migration, and at st.27 (24 somite stage) form bilateral clusters in the ventrolateral areas of the trunk at the areas immediately anterior to the separation of the trunk and yolk (Fig 1C, bracket) [26, 27, 44, 45]. Thereafter, the PGCs enter the medial region (Fig 1E, bracket) at st. 31 (4 days post fertilization), then they move to the dorsal mesentery where the gonads eventually develop at st. 34 (5 days embryo) [29, 46–49].

We identified a recessive mutation, *naruto*^*j113-2B*^ (*nar*^*j113-2B*^), which affects the PGC migration pattern, through a mutant screening focusing on early PGC development [20]. In the *nar* homozygous mutant embryo, PGCs line up bilaterally beside the posterior trunk with a distribution that is indistinguishable from the wild type at st.22 (Fig 1B, bracket). But then, PGCs fail to make a posterior migration and scatter on the anterior side of the yolk sacs lateral to the normal position of the PGC clusters at st.27 (Fig 1D, dashed line). Subsequently, at st. 31, PGCs are distributed in the ectopic area and a part of PGCs gather in the presumptive gonad area (Fig 1F). Because the total number of *olvas* positive cells tends to be lower in this stage (data not shown), we can infer that PGCs ectopically distributed outside of the gonad area may die or lose *olvas* expression.

The *nar* mutation causes other characteristic pleiotropic defects including abnormal distribution of posterior lateral line primordia and various morphogenetic defects. The posterior lateral line primordia migrate posteriorly on horizontal myoseptum in wild-type embryos at st.27 [26, 35], but in the mutant embryos fail to make a posterior migration and are distributed further to the lateral side compared the normal migrating path in wild-type embryos (Fig 1G and 1H). In addition, the *nar* homozygous embryos showed various morphological abnormalities including enlarged brain ventricles at st.27 (Fig 1I–1L), and curved trunks, enlarged pericardial spaces and thin hearts at st. 35 (Fig 1M–1P). The blood circulation starts but then decreases, and stops around at st.27 with blood cells accumulating in the embryo (Fig 1N). The *nar* homozygous embryos die before hatching with these systemic defects.

### Mutation in *cpsf6* gene is responsible for the *nar*^*j113-2B*^ phenotype

The *nar*^*j113-2B*^ was mapped between the *erc1* and the *smo* loci on chromosome 23 in a genetic linkage mapping using 1716 recombination density F2 panel (Fig 2A). Further fine mapping using 5 or 9 recombinants at the *erc1* or the *smo* loci, respectively, resulted in narrowing down (Fig 2A, horizontal black arrows) the *nar* candidate locus to within a 33kb region (Fig 2A, double-headed horizontal red-arrow) containing an ORF of the *cpsf6* and 5' region of *cacna2d4*, excluding other genes (e.g., *prr5*, *napepld*, *yeats4*, *dcp1b* and *rtm2*) from mutant gene candidates. Genomic DNA sequencing covering all exons in the ORF of the *cpsf6* revealed T to A mutation at a splice donor site of exon 2 in the *nar*^*j113-2B*^ mutants (Fig 2B). The cDNA in this region was analyzed and we confirmed the intron between exon 2 and 3 does not splice out in the transcript in the *nar* mutant embryo (Fig 2D and 2E and Materials and methods). This intron insertion caused by the *nar*^*j113-2B*^ mutation in the *cpsf6* transcripts is predicted to result in an additional seven amino acids terminated with a stop codon after W^90^ in the expected CPSF6 amino acid sequence in the *nar* mutant embryos (Fig 2B). Because of the complete loss of conserved domains required for interaction with CPSF5/CFIm25, binding with RNA, and subcellular localization (Fig 2C) [50, 51], the mutated CPSF6 protein is expected to lose almost all function. The mutation in the *nar*^*j113-2B*^ genomic DNA also created a new *MnlI* restriction site (Fig 2B), which was subsequently utilized in the genotyping of this allele (Materials and methods).

Whole-mount *in situ* hybridization using a *cpsf6* antisense probe indicates the *cpsf6* is expressed ubiquitously in the medaka embryo at st.22 and 27 in the wild-type embryos (Fig 2F and 2H). In contrast, this expression was extremely reduced in the *nar* mutant embryo at st.27 (Fig 2H and 2I). This reduction of the *cpsf6* expression in the *nar* mutant embryos could be a result of nonsense-mediated mRNA decay [52, 53].

**Fig 3:**
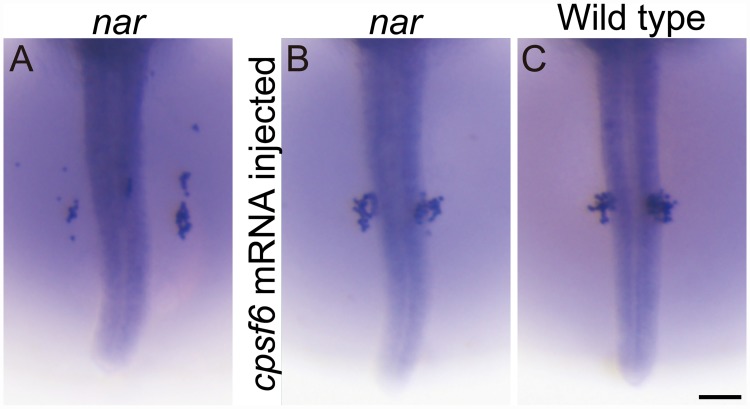
Rescue of the *nar*^*j113-2B*^ phenotype in the PGC distribution by embryonic injection of the *cpsf6* mRNA. (A-C) Whole mount *in situ* hybridization using the *olvas* probe of same clutch embryos at st. 27. Dorsal views are shown in the orientation of anterior toward the top. PGCs are scattered on the yolk sac more anterior and lateral to the normal clustering position in the *nar*^*j113-2B*^ homozygous embryo (A). The wild-type (B) and the *nar*^*j113-2B*^ homozygous (C) embryos were injected with the full length *cpsf6* mRNA with the short 3'UTR at 1 cell stage. The *cpsf6* mRNA injection does not affect the PGC positioning to the normal clustering position in the wild-type embryo (B). In the *nar*^*j113-2B*^ homozygous embryo injected with the *cpsf6* mRNA (C), PGCs are clustered in the normal position similar to wild type (B) at st. 27. Scale bar, 100 μm.

To confirm that the mutation in the *cpsf6* is responsible for the *nar*^*j113-2B*^ homozygous phenotype, *cpsf6* mRNAs were synthesized from full length cDNA clones with 587b or 2004b 3'UTR, or the *cpsf6* ORF inserted into a *pCS2+* vector [41, 42] and injected into fertilized eggs obtained from the *nar*^*j113-2B*^ heterozygote crosses. All the injected embryos were developed and sorted by morphology at st.27, we then checked the PGC distribution by *in situ* hybridization by labeling the *olvas* probe, photographed, and genotyped the *nar*^*j113-2B*^ mutation (Table 1). More than 60% (28/42) of the *nar*^*j113-2B*^ homozygous embryos injected with the full-length *cpsf6* mRNAs (both those with short (587b) and long (2004b) 3'UTRs) showed normal morphological shape and normal PGC distribution (Fig 3C) in the embryos that developed without severe malformation until st.27. In addition, the other homozygous mutant embryos injected with the mRNAs showed a morphologically *naruto* homozygote phenotype, however, they possessed a normal PGC distribution. In contrast, all of the *nar*^*j113-2B*^ homozygous embryos injected with mRNA synthesized from the *cpsf6* ORF inserted into a *pCS2+* vector showed the *naruto* morphological phenotype, however, the distributions of the PGCs were indistinguishable from that of the wild-type embryos. Injection of the synthetic mRNAs to wild-type eggs did not have any appreciable effect (Fig 3B). The partial rescue effect using mRNA without native 3'UTR is expected to require an adequate posttranscriptional regulation mediated by the 3'UTR to show full rescue effects. These results, that wild-type *cpsf6* overexpression efficiently rescued the *nar*^*j113-2B*^ homozygous phenotypes using several kinds of mRNA constructs, clearly show the gene responsible for the *nar*^*j113-2B*^ is *cpsf6*.

**Table 1:** Number of embryos injected with the *cpsf6* mRNA.

Injected mRNA	Morphology^a^					
	Normal^b^		*naruto* shape		Malformed	
	Genotype		Genotype		Genotype	
	*nar*^*+/+*, *+/-*^	*nar*^*-/-*^	*nar*^*+/+*, *+/-*^	*nar*^*-/-*^	*nar*^*+/+*, *+/-*^	*nar*^*-/-*^
*cpsf6 short 3'UTR*	110		10		83	
	95	15	0	10^c^	61	22
*cpsf6 long 3'UTR*	78		4		28	
	65	13	0	4^c^	21	7
*cpsf6 ORF*	73		24		20	
	73	0	0	24^c^	16	4
not injected	35		11		18	
	35	0	0	11^d^	14	4

^a^The mRNA injected embryos were developed and sorted by morphology at st. 27 with normal, *naruto* or malformed shapes.

^b^The distributions of the PGCs in the all embryos with normal morphology were indistinguishable from that of the wild-type embryos.

^c^The embryos show morphologically *naruto* homozygote phenotype, however, the distributions of the PGCs were indistinguishable from that of the wild-type embryos.

^d^All embryos showed *naruto* homozygote phenotype in terms of morphology and PGC distribution.

### Poly A additions occurred aberrantly upstream and caused short 3'UTRs in the *nar*^*j113-2B*^ mutant embryos

**Fig 4:**
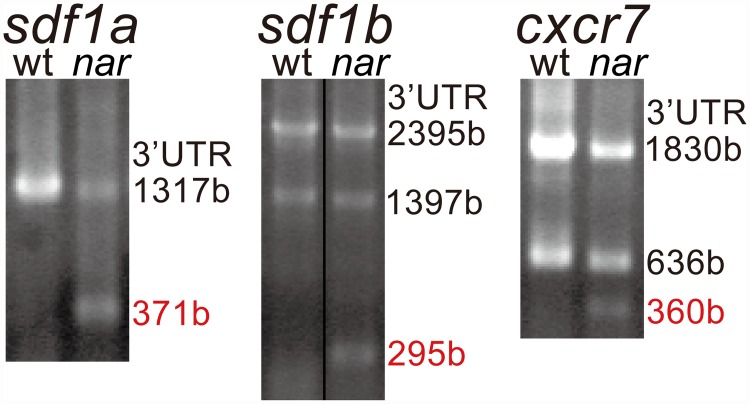
Poly A addition occurred unusually upstream and that caused short 3'UTRs in the *nar* mutant embryos. 3' UTRs of the *sdf1a*, *sdf1b* and *cxcr7* were amplified by 3' RACE PCR from the total RNA of wild-type (wt) and *nar*^*j113-2B*^ homozygote (*nar*) embryos at st. 27, and separated by agarose gel electrophoresis. Short 3'UTRs (red) are detected in the *nar* mutant embryos in addition to 3'UTRs the same length as the wild type. The lengths of the 3'UTRs were determined by sequencing of cDNAs purified from each excised band. Actual sizes of each band are as follows, *sdf1a*: 1500, 600bp; *sdf1b*: 2600, 1600, 550bp; *cxcr7*: 2000, 800, 500bp. Upper and lower ends of the gel-images correspond to 4kb and 400bp respectively. Concentration of the genomic-DNA templates is not adjusted equally between the wild-type and mutant embryos.

CPSF6 is one component of the Cleavage Factor Im complex (CFIm) that plays a key role in pre-mRNA 3'-processing and is involved in deciding the cleavage point in the 3' end cleavage and polyadenylation processing by recognizing the UGUA sequence existing upstream of the polyadenylation signal [54, 55]. Therefore, this process was expected to be affected in the *naruto* mutant embryo. The fact that the *naruto* homozygote embryos have defects in the posterior migration of PGCs and posterior lateral line primordia (Fig 1D and 1H) suggest aberrations in the Cxcr4 and Cxcr7 mediated Sdf1 chemokine signaling that is known to be commonly involved in the guidance of these two cell types [7, 26, 56–61]. Because previous study indicates a mutation in *cxcr4b* has effects from an earlier stage than st.22 with more severe defects in PGC migration [26], it is expected that the gene expression of the *sdf1a*, *sdf1b*, and *cxcr7*, expressed in the surrounding mesodermal tissues of posterior migrating PGCs is affected in the mutant embryo. 3'RACE (Rapid Amplification of cDNA 3' Ends) analysis for these genes showed, indeed, an extra short band compared to wild type was found in each gene at st.27 (Fig 4). Sequencing of the 3' end region of all the distinct bands indicated poly-A additions occurring in more upstream sites than those in the wild-type embryos resulted in the extra transcripts with short 3'UTRs in all the gene cases (Table 2).

**Table 2:** The 3' end 100b sequences of 3'UTRs amplified by 3'RACE.

Gene	Length^a^	Sequence^b^
*sdf1a*	1317b	AUAUGAAGGAAC**UGUA**UACUUUCACAACUGAGCCAUCCUGCUUUUUUU**UGUA**AACC**UGUA**UCACAGCUGCUGAGUAAAAACAAGACAUUUUUAAAAGUCA
	371b*	CUUUUUUUAGUAGUUGUCGAUAGAUUUUAACAGUAAAGCAACAUUUUUGAAGCCAGAGUUGUUCUGGCAGGCCUGUUUAUCAAUUAAAAAAA**UGUA**UACA
*sdf1b*	2395b	UUUCUUUGUCACUUUU**UGUA**UUCUCACUGGU**UGUA**AUCCAAGAGGCAGUGUUUUAGAGAUUUAUAUAUAUGCUCAUUUUUCAAUAAAGGCAUUUAAAACA
	1397b	AUACAUGAAUCUAUCAUUUUAUAAAUGCUUUAAA**UGUA**AUGUGU**UGUA**GACAGUAAAUGAAGUUAUCAAACUGCAGUAUUAAACUUCUAUAUUCCUUUCA
	295b*	GGAAAACUUGACAUCCCGUGUCCACAUGUGUGUGUCUUUAUGGACACAUCAGUGCAGGAGCAGGCCAGCUUCC**UGUA**AAAGAAAAAUAUGUUCCCCCUGA
*cxcr7*	1830b	CAAAGUGAGAGAAAACAAACAUGUUUCUACAAGAACAGAGAAGUUCUUUUGUUAU**UGUA**A**UGUA**UUACCUGUUUAAUUACAUAAUAAACACAGAAAAAUG
	636b	UCUCUUAAAGGCAAUAACCUUUUUCUUCCAAGGAAUGUUUUUUUUCAUGGAUUGAUUUACAUAAAGAUGUGAGACU**UGUA**UGCAUAAAUUUUAAUGUUUA
	360b*	GUUUGAA**UGUA**UUUUGUUUGCAUUUUU**UGUA**UGAGGUCUGAUGUCGUACCAGGAG**UGUA**AUAUCUU**UGUA**UUUAGU**UGUA**UUUAUAUUCAUUUGUGUGGA

^a^Total length of the 3'UTR for the 3'RACE amplified products (Fig 4). Asterisks (*) indicate the short 3'UTRs amplified specifically in the *naruto* mutant embryos.

^b^RNA sequence 100b upstream from the distal end of poly A in each transcript. Bold and underline: The signal sequence recognized by the CFIm complex (UGUA). Double underline: Canonical polyadenylation signals (AAUAAA, AUUAAA). Underline: Non canonical polyadenylation signals (AGUAAA, UAUAAA, CAUAAA, GAUAAA, AAUAUA, AAUACA, AAUAGA, ACUAAA, AAGAAA, AAUGAA) (Beaudoing et al., 2000; Kamasawa and Horiuchi, 2008).

### Transcript distribution of *sdf1a* and *sdf1b* in the *naruto* mutant somite is aberrantly extended to the anterior side at the PGC migrating stage

The 3'UTR of mRNA contains *cis*-regulatory elements targeted by miRNAs and RNA-binding proteins and has important roles in post-transcriptional gene regulation involving the regulation of mRNA stability and translational control [31–33]. That the short 3'UTRs caused altered transcript distribution in the mutant embryos was observed via whole mount *in situ* hybridization using an antisense probe designed specifically for the full-length ORF and distal 3'UTRs in *sdf1a*, *sdf1b*, or *cxcr7*. At st.22, no difference in the transcript distribution was observed between the wild-type and the mutant embryos in any cases (S1 Fig).

**Fig 5:**
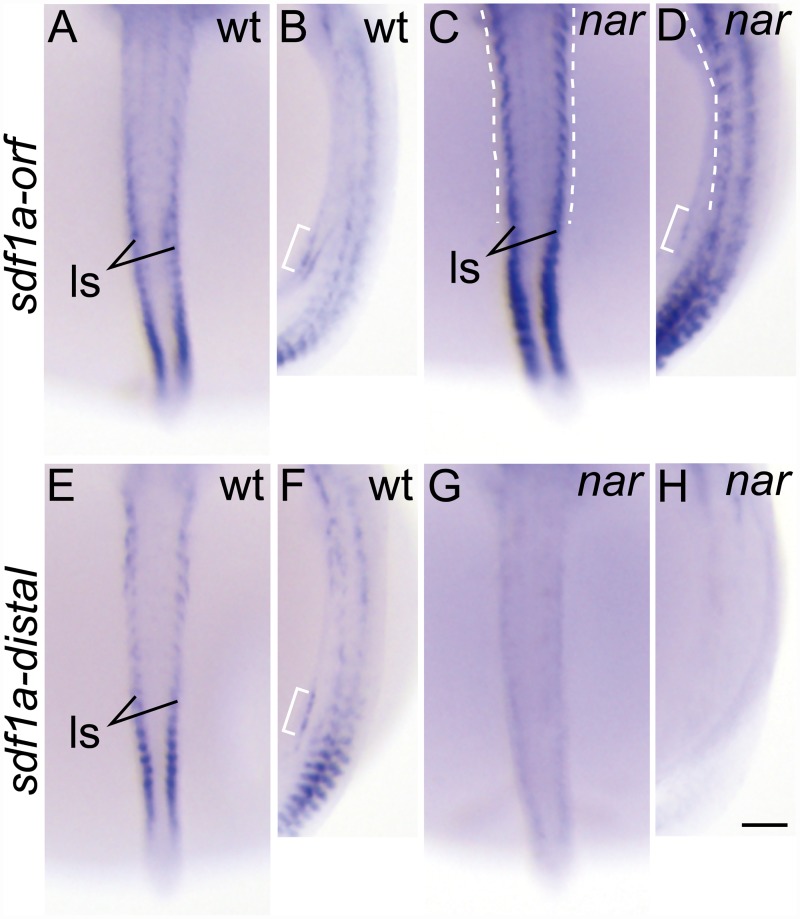
The transcript distribution of the chemokine *sdf1a* is anteriorly extended in the *nar* mutant somite at st. 27. (A-H) Whole mount *in situ* hybridization using an antisense probe specific to the *sdf1a* ORF (A-D, *sdf1a-orf*) or distal 3'UTR (E-H, *sdf1a-distal*) at st. 27. Wild-type (A, B, E, F) and *nar* homozygote (C, D, G, H) embryos were stained using each probe. The *sdf1a* signals are observed in the mesodermal tissue corresponding to the gathering PGC region (B, D, F, bracket). The *sdf1a* signals in the ventrolateral somites (ls) are extended more to the anterior side than that in the wild type in the *nar* mutant embryos (C, D, dashed lines) using the ORF specific probe. The *sdf1a* signal is hardly detected in the *nar* mutant embryo using an *sdf1a* distal-3'UTR specific probe (G, H). Dorsal (A, C, E, G) and the lateral (B, D, F, H) views are shown in the orientation of anterior toward the top. AB, CD, EF, GH are the same embryos. Scale bars indicate 100 μm.

**Fig 6:**
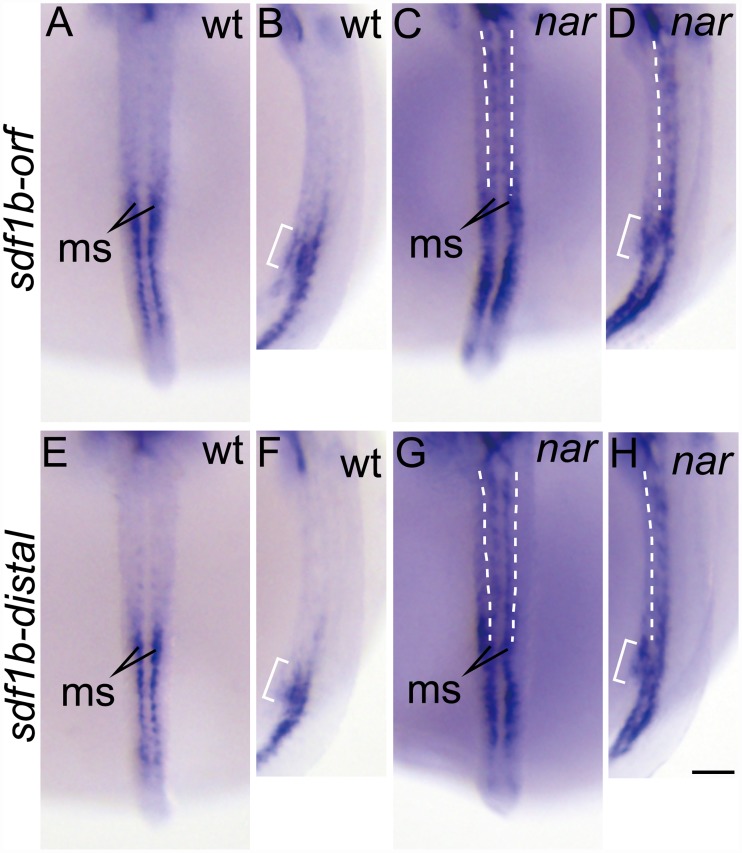
The transcript distribution of the chemokine *sdf1b* is anteriorly extended in the *nar* mutant somite at st. 27. (A-H) Whole mount *in situ* hybridization using an antisense probe specific to the *sdf1b* ORF (A-D, *sdf1b-orf*) or distal 3'UTR (E-H, *sdf1b-distal*) at st. 27. Wild-type (A, B, E, F) and *nar* homozygote (C, D, G, H) embryos were stained using each probe. The *sdf1b* transcript distributions are observed in the mesodermal tissue corresponding to the gathering PGC region (Fig 5B, 5D and 5F, bracket) more medial to the *sdf1a* signals (B, D, F, H, bracket). The *sdf1b* signals in the ventromedial somite (ms) are extended more to the anterior side than that in the wild type in the *nar* mutant embryos using ORF (C, D, dashed lines) or distal 3'UTR (G, H, dashed lines) specific probes. Dorsal (A, C, E, G) and the lateral (B, D, F, H) views are shown in the orientation of anterior toward the top. AB, CD, EF, GH are the same embryos. Scale bars indicate 100 μm.

In the wild-type embryos at st.27, an *sdf1a* signal was detected, using an ORF specific probe, in the mesodermal tissue corresponding to the PGC gathering region (Fig 5B, bracket) and ventrolateral somite with that was weaker to the anterior side and stronger to the posterior side (Fig 5A, ls). Surprisingly, in the *nar* mutant embryos, the strong signal in the ventrolateral somite was extended more to the anterior side than that in the wild type (Fig 5C and 5D, dashed lines). By using an *sdf1a* distal-3'UTR specific probe, a strong signal was located in the posterior side (Fig 5E and 5F), which was the same as when using the ORF specific probe in wild-type embryos. On the other hand, the signal was almost undetected in the *nar* mutant embryo (Fig 5G and 5H). The *sdf1b* mRNA signal in the *nar* mutant embryos was extended more to the anterior side in the ventromedial somite (Fig 6C, 6D, 6G and 6H, dashed lines) than that in the wild-type embryos (Fig 6A, 6B, 6E and 6F) at st.27 by using both type of probes, ORF (Fig 6A–6D) and distal (Fig 6E–6H) 3'UTR specific probes.

In the *cxcr7* transcript distribution in the tissues surrounding the migrating PGC route at st.22 and st.27 embryos, using both ORF and distal 3’UTR specific probes, no difference was observed between in the wild type and the *nar* mutant (S1I–S1L and S3 Figs).

## Discussion

### Aberrant poly A site selection caused by the *cpsf6* mutation in the developing embryos

In this study, we focused on *naruto*, a recessive homozygous lethal mutation affecting PGC migration. A fine genetic linkage mapping revealed the responsible gene is *cpsf6*. CPSF6 is a component of the CFIm complex that plays a key role in pre-mRNA 3'-processing and is required for 3' RNA cleavage and polyadenylation processing. 3'RACE in the mutant embryos showed the poly A additions occurred upstream and that caused short 3'UTRs in the chemokine and its receptor genes, *cxcl12/sdf1* and *cxcr7*, which is known to be involved in PGC migration (Fig 4, Table 2).

The 3'-ends of transcripts contain a polyadenylation signal, A(A/U)UAAA hexamer or a variant, in 10–30 nucleotides upstream of the cleavage site, and one or more UGUA sites a variable distance upstream of the polyadenylation signal in most cases in metazoa [55, 62, 63]. In this study, both of the typical signal sequences were found in the 3'-end regions in the transcripts that were commonly seen in the wild-type and mutant embryos at st.27. However, sets of the signal sequences were not found upstream of the 3'-end regions in the extra short 3' UTRs in the mutant embryos, except in the case of *sdf1b* (Table 2). This suggests the 5' upper sites in UTRs, which are normally not selected, are selected as poly A addition sites in the *naruto* mutant embryos. This may support the idea that the CFIm could have a role in the regulation of the poly A site selection by suppressing the cleavage of the pre-mRNA [64, 65].

In this study, polyadenylation sites were analyzed in the transcripts focused on chemokine and its receptor genes: *sdf1a*, *sdf1b* and *cxcr7*, in the *nar* mutant embryos at the PGC migration stages. Because the CFIm complex constructed from the CPSF6 widely contributes to the metazoan poly A site selection [55], the *nar* mutation would affect the 3'UTR length and stability of the transcripts in a wide variety of genes. This might be the reason that the mutant dies before hatching and exhibits systemic defects in a wide variety of tissues. However, the morphogenesis proceeds to a relatively late stage in the mutant embryos, even with abnormal morphology. This might result from the fact that not only extra mRNAs with abnormal short 3'UTRs, but also mRNAs with normal length 3'UTRs, are expressed in the mutant embryo. Especially, in the case that the length of the extra short 3'UTRs synthesized by the defect in the CPSF6 are relatively similar with that of the wild-type 3'UTRs, it is suggested that, like in *cxcr7* ‘s case (Fig 4), the extra transcripts with short 3'UTRs do not have a severe effect on the embryonic development as a result of how few 3’UTR sequences are lost. Despite the mutation in *cpsf6*, normal poly A sites are also selected in the mutant embryos. This might support the suggestion that the CPSF6 and CPSF5/CFIm25 might function redundantly by forming heterotetramers with CPSF5/CFIm25 dimer in the CFIm complex [51, 66]. It is possible that CPSF6 contributes to the poly A site selection in other genes as well, which causes morphological abnormality of the mutant, an issue to be explored in the future.

Previous studies suggest that the CFIm complex has a regulatory role in alternative poly A site selection [51, 65–69]. A large portion of eukaryotic genes have multiple mRNA isoforms that differ in the length of the 3'UTRs by the alternative polyadenylation, therefore this will affect the stability of the transcripts and the translational control [32, 70]. In addition, recent studies have revealed that the misregulation of the alternative polyadenylation contributes to the development of several human diseases including cancer [32, 71, 72]. Using the *nar* mutant embryos in future research to analyze whether and how CPSF6 contributes to the alternative polyadenylation of various genes in a tissue and developmental stage-specific manner will provide new and intriguing insights. In addition, we believe this mutant will prove to be an important tool in studying the involvement of 3'UTRs in posttranscriptional gene regulations in various genes *in vivo*.

### Possible mechanisms of the defects in the PGC migration in the *nar*^*j113-2B*^ mutant embryos

Whether mRNAs with abnormally short 3'UTR alter the transcript distributions of the chemokine and its receptor genes, *sdf1a*, *sdf1b* and *cxcr7* was observed in the *nar* mutant embryos at the PGC migration stage. The result shows the *sdf1a* mRNA with a short 3'UTR is dominantly expressed in the *nar* mutant embryos, with the expression region in the ventrolateral somite extended further to the anterior side than in the wild type at st.27 (Fig 5). In the wild-type embryos, *sdf1a* mRNA would be immediately eliminated by the post-transcriptional degradation system mediated by the miRNAs and/or RNA binding proteins in the anterior ventrolateral somite area. Whereas in the *nar* mutant embryos, *sdf1a* mRNA with short 3'UTRs, with their possible lack of target sites for the miRNA and/or RNA binding proteins in the distal 3'UTR, may escape from the degradation system. Whether possible targets for the 146 medaka miRNAs registered in the miRNA database (miRBase, http://www.mirbase.org/)[73] exist in the 3'UTR was predicted using the RNAhybrid program (ver.2.2, http://bibiserv.techfak.uni-bielefeld.de/rnahybrid)[74, 75]. As a result, 15 miRNAs were selected with the conditions that the seed region, nucleotides 2–7 of the miRNAs, matches completely to the target sites and that the minimum free energy of the hybridization is less than -20 kcal/mol [76, 77] (S2 Fig). Of these miRNAs, 10 miRNAs have target sites in the distal region that is not present in the short 3'UTR. In addition, these 10 miRNAs contained miR-430. This miRNA is suggested to be involved in the regulation of the *sdf1a* mRNA stability in zebrafish embryos [4–6]. Future studies into which of these candidate miRNAs really regulate the stability of the *sdf1a* mRNA in medaka embryos could yield results of great interest.

The *sdf1b* mRNA signal in the ventromedial somite was extended more to the anterior side in the *nar* mutant embryos than that in the wild type (Fig 6). However, in contrast to the case in *sdf1a*, the expressed *sdf1b* mRNA in the ectopically anterior ventromedial somite includes at least the normal *sdf1b* mRNA with long 3'UTR, although the expression area of the *sdf1b* mRNA with the short 3'UTR could not be specified (Fig 6). This may suggest that the malfunction of the CPSF6 in the *nar* mutant embryo may cause the production of mRNAs with abnormally short 3'UTR in multiple genes, which may affect the gene expression involved in the transcriptional or posttranscriptional regulation of the *sdf1b* gene.

No difference between in the wild type and the *nar* mutant was observed in the *cxcr7* transcript distribution at the PGC migration stage (S1 and S3 Figs). In the *cxcr7*, the difference in length between the short 3'UTR in the *nar* mutant and the normal 3'UTRs is relatively small in comparison with that in *sdf1a* and *sdf1b* (Fig 4). Therefore, the *cxcr7* mRNA with the short 3'UTR expressed in the mutant lost only a small part of the sequences. This may result in no effects from the expression of the short 3'UTR.

The PGCs originating from the animal pole-proximal region of the blastoderm at the early gastrula stage move to the marginal zone of the embryonic shield at the mid-gastrula stage, then the posterior region of the embryonic body at early-neurula stage, finally forming bilateral lines along the body axis in the posterior lateral plate mesoderm that expresses *sdf1a* and *sdf1b* at st.22 (Fig 1A, S1A and S1E Fig) [26–29]. This PGC translocation is thought to result from integrated sequential movement through both the active chemotactic migration of CXCR4b expressing PGCs responding to the gradient of the chemoattractant signals of SDF1a and SDF1b, and also by passive movement forced by the surrounding cells [27, 28]. PGCs thereafter migrate in the lateral plate mesoderm to the posterior side; finally gathering in the ventrolateral areas of the prospective gonad at st.27 (Fig 1C). The chemokine signal gradients formed in the somatic environment are thought to also be critical to this PGC posterior migration. It is thought that the localization of *sdf1a* and *sdf1b* to the posterior side by regulation of gene expression in the PGC migration stage, and clearing of the SDF1 proteins from the somatic environments by CXCR7, which is a decoy receptor for SDF1 are vital in the formation of the posterior dense SDF1 signal gradients [7, 8, 26, 27, 29]. The SDF1 gradients would be disturbed by the extra expression of the *sdf1a* and *sdf1b* in the anterior mesodermal tissues in the *nar* mutant embryos at st.22-27. The loss of accurate directional information would be the reason for the defect in the posterior migration of the PGCs in the mutant embryos. Analysis of the SDF1 protein expressions in the *nar* mutant embryos via immunohistochemistry, and analysis of the influence of the 3'UTR length on the transcript levels and protein turnover *in vivo* should be addressed in future research.

In the posterior lateral-line development, it is indicated that the lateral-line primordia expressing the *cxcr4b* are guided by the *sdf1a* signal gradients formed in the migration route, with the primordia towing the lateral-line nerves [26, 35, 78, 79]. Therefore, the defect in the posterior migration of the lateral-line primordia in the *nar* mutant embryos (Fig 1H) is also suggested to result from the disturbance in directional guidance caused by the strong *sdf1a* expression in the anterior somite area (Fig 5C and 5D), the same as in the case of PGC-migration.

## Supporting information

S1 Fig Whole mount *in situ* hybridization using an antisense probe specific to the ORF or distal 3'UTRs in *sdf1a*, *sdf1b* or *cxcr7* at st. 22. Wild-type (A, C, E, G, I, K) and *nar* homozygote (B, D, F, H, J, L) embryos were stained using each probe. At st.22, no difference in the transcript distribution is observed between the wild-type and the *nar* mutant embryos in all cases. (A-D) The *sdf1a* signals are detected using an ORF (A, B, *sdf1a-orf*) or distal 3'UTR (C, D, *sdf1a-distal*) specific antisense probe. No difference is observed in the transcript distribution (A-D). (E-H) The *sdf1b* signals are detected using an ORF (E, F, *sdf1b-orf*) or distal 3'UTR (G, H, *sdf1b-distal*) specific antisense probe. No differences are observed in the transcript distribution (E-H). (I-L) The *cxcr7* signals are detected using an ORF (I, J, *cxcr7-orf*) or distal 3'UTR (K, L, *cxcr7-distal*) specific antisense probe. Weak signals are detected using the distal 3'UTR specific antisense probe (K, L) then comparing it with the ORF specific antisense probe (I-L). The *nar* homozygote embryos were identified by genotyping to detect the *nar*-mutation allele in each embryo after *in situ* hybridization and taking photographs (see "Genotyping for *nar* mutation" in the Materials and methods section). Dorsal views are shown in the orientation of anterior toward the top. All embryos are flat-mounted. lp, lateral plate mesoderm; psm, presomitic mesoderm; sc, spinal cord; sm, somites. Scale bars indicate 100 μm.
(TIF)

S2 Fig Possible miRNA bindings in the *sdf1a* 3'UTR. A. A 1,317b RNA sequence in the *sdf1a* 3' UTR. The red triangle shows the end of the 371b length of the short 3' UTR found in the *nar* mutant embryos. Red letters and underlined regions 1–9 show predicted targets for the seed regions of the miRNAs registered in the miRNA database. B-J. Predicted secondary structures of the miRNA/target duplexes in the target regions 1–9 shown in A. the green sequences are miRNAs and the red sequences are the target 3'UTRs. Minimum free-energy (mfe) values of the hybridizations and the names of the miRNAs are shown in the figures.
(PDF)

S3 Fig Whole mount *in situ* hybridization using an antisense probe specific to the ORF or distal 3'UTRs in *cxcr7* at st. 27. *cxcr7* transcript distribution is detected strongly in the dorsal part of the somite (ds), the ventral part of the somite (vs), and the spinal cord (sc) using an antisense probe specific to the ORF (A-D, *cxcr7-orf*) in wild-type (A, C) and *nar* homozygote (B, D) embryos, though the signals in the spinal cord are weaker in the mutant embryos. Weak signals are detected using the distal 3'UTR specific antisense probe (E-H, *cxcr7-distal*) then they are compared with the ORF specific antisense probe (A-D). (A, B, E, F) Dorsal views are shown in the orientation of anterior toward the top. Lateral views of A, B, E, F are shown in C, D, G, H in the orientation of anterior toward the left. Scale bars indicate 100 μm.
(TIF)

S1 Table Primer information used in the genetic linkage mapping. Primer information used in the genetic linkage mapping of the *nar*^*j113-2B*^ mutation.
(DOCX)
