# Supplementary figures and images for: Mutation in cpsf6/CFIm68 (Cleavage and Polyadenylation Specificity Factor Subunit 6) causes short 3'UTRs and disturbs gene expression in developing embryos, as revealed by an analysis of primordial germ cell migration using the medaka mutant naruto
Source: PLoS One. 2017 Mar 2;12(3):e0172467. doi: 10.1371/journal.pone.0172467 (PMC5333813; doi:10.1371/journal.pone.0172467)

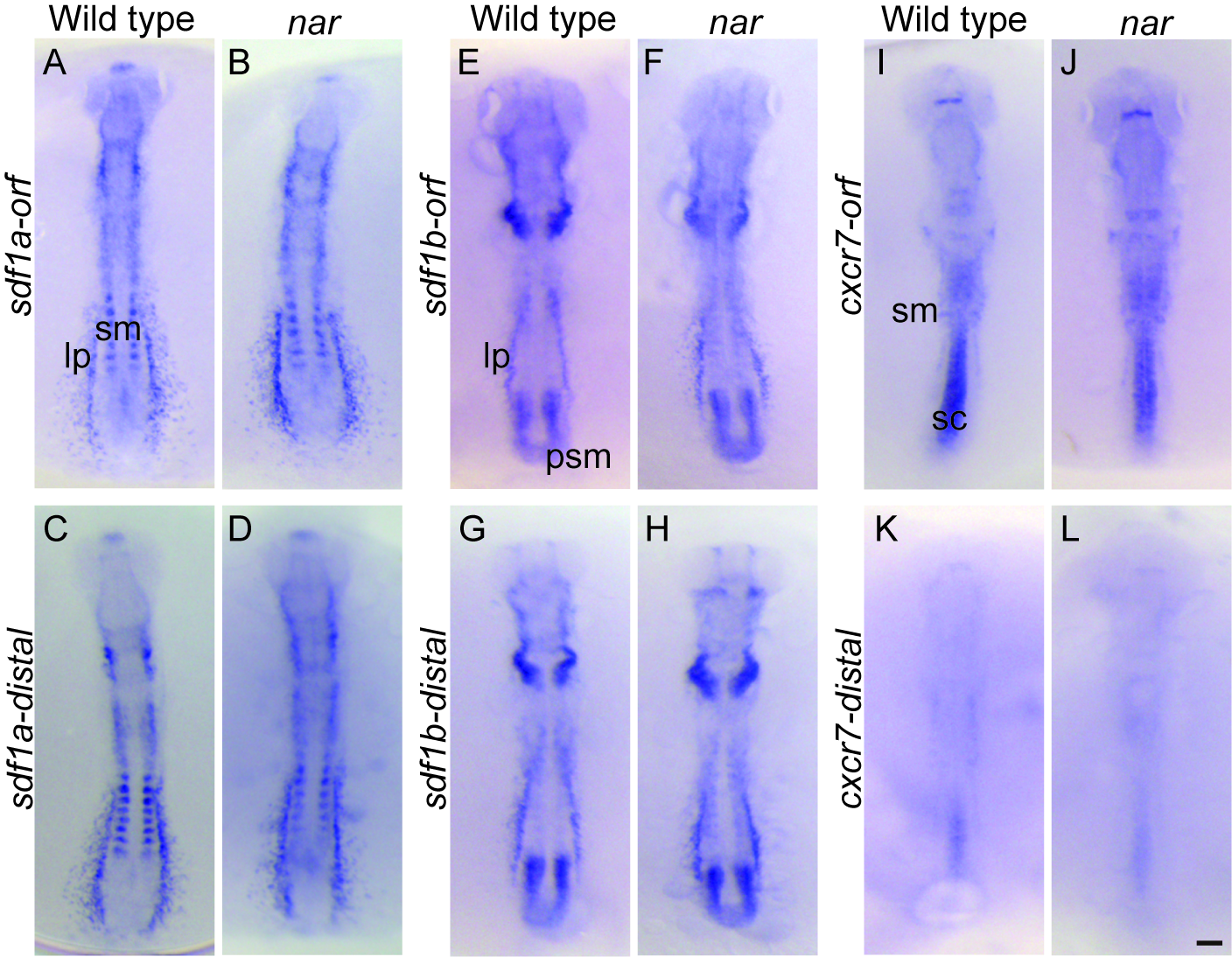

Supplement: S1 Fig — Wild-type (A, C, E, G, I, K) and nar homozygote (B, D, F, H, J, L) embryos were stained using each probe. At st.22, no difference in the transcript distribution is observed between the wild-type and the nar mutant embryos in all cases. (A-D) The sdf1a signals are detected using an ORF (A, B, sdf1a-orf) or distal 3'UTR (C, D, sdf1a-distal) specific antisense probe. No difference is observed in the transcript distribution (A-D). (E-H) The sdf1b signals are detected using an ORF (E, F, sdf1b-orf) or distal 3'UTR (G, H, sdf1b-distal) specific antisense probe. No differences are observed in the transcript distribution (E-H). (I-L) The cxcr7 signals are detected using an ORF (I, J, cxcr7-orf) or distal 3'UTR (K, L, cxcr7-distal) specific antisense probe. Weak signals are detected using the distal 3'UTR specific antisense probe (K, L) then comparing it with the ORF specific antisense probe (I-L). The nar homozygote embryos were identified by genotyping to detect the nar-mutation allele in each embryo after in situ hybridization and taking photographs (see "Genotyping for nar mutation" in the Materials and methods section). Dorsal views are shown in the orientation of anterior toward the top. All embryos are flat-mounted. lp, lateral plate mesoderm; psm, presomitic mesoderm; sc, spinal cord; sm, somites. Scale bars indicate 100 μm. (TIF) [file pone.0172467.s001.tif]

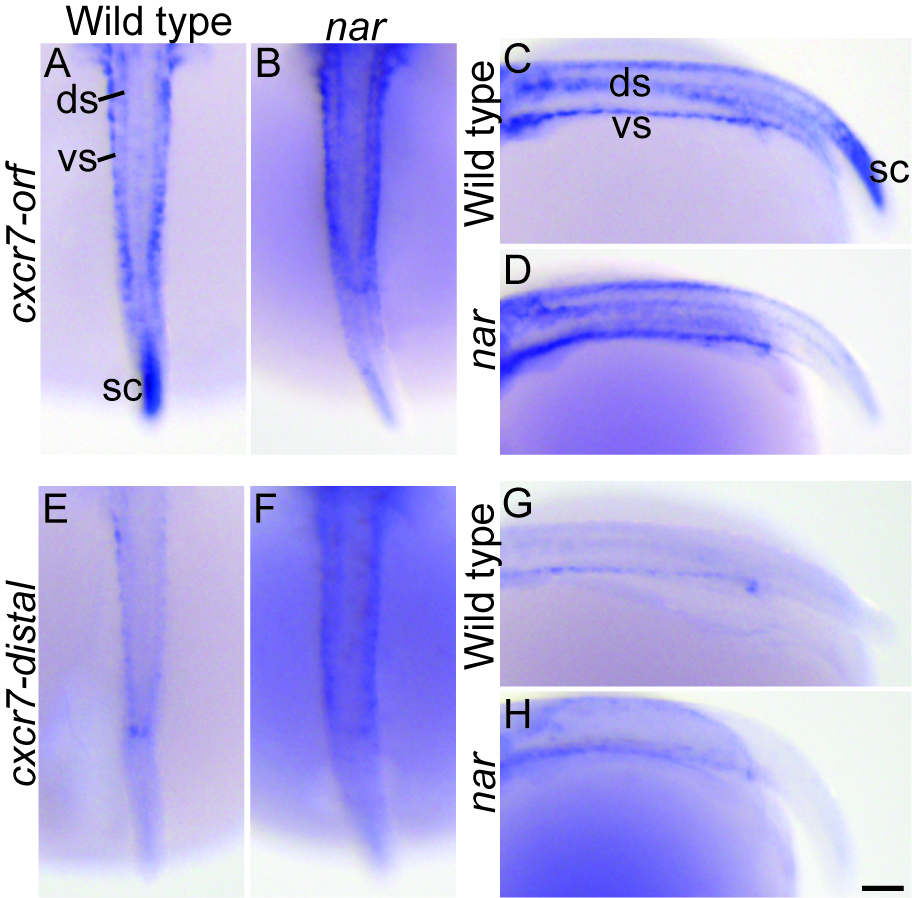

Supplement: S3 Fig — cxcr7 transcript distribution is detected strongly in the dorsal part of the somite (ds), the ventral part of the somite (vs), and the spinal cord (sc) using an antisense probe specific to the ORF (A-D, cxcr7-orf) in wild-type (A, C) and nar homozygote (B, D) embryos, though the signals in the spinal cord are weaker in the mutant embryos. Weak signals are detected using the distal 3'UTR specific antisense probe (E-H, cxcr7-distal) then they are compared with the ORF specific antisense probe (A-D). (A, B, E, F) Dorsal views are shown in the orientation of anterior toward the top. Lateral views of A, B, E, F are shown in C, D, G, H in the orientation of anterior toward the left. Scale bars indicate 100 μm. (TIF) [file pone.0172467.s003.tif]
