# Supplementary material for: Mutation in cpsf6/CFIm68 (Cleavage and Polyadenylation Specificity Factor Subunit 6) causes short 3'UTRs and disturbs gene expression in developing embryos, as revealed by an analysis of primordial germ cell migration using the medaka mutant naruto
Source: PLoS One. 2017 Mar 2;12(3):e0172467. doi: 10.1371/journal.pone.0172467 (PMC5333813; doi:10.1371/journal.pone.0172467)

A

sdf1a mRNA 3’ UTR

AAGGCCUUAGAGAUGCUCCUAAGCUGGGGGCAUCUGGAGCAUCGCCAUGGAUAUGUAACGUGAGAUCGUCU
GGCGUGUUACACAGCACUUAUACCAGCUGCCGUCCUCUAGCCUCGUGCACGGACUGAACUGCAACUCCUCA
1 2 3
CCAUGAACCCCCGUCCACCUAACCUCCUGCCGUCUUCACCAUCACCACUACCACUUCUUAUAUCAAAAAAUA
UUAUUGAAAAGUGGCAUUAUACCUGCGAGUGUAUGUUUUCUAACCAUUCUGUACAUACUUUUUUUAGUAGUU
GUCGAUAGAUUUUAACAGUAAAGCAACAUUUUUGAAGCCAGAGUUGUUCUGGCAGGCCUGUUUAUCAAUUAA
AAAAAUGUAUACAAAUUUUGCUUAAAUUUCCUAAAAGGGAAGAUAUGAGUCAAAGCAUUCUGCUCUGCUCAA
UGUUUUUGAAAACUAAACAGAAAAUCUGCUCAUAUUAAUUUUCUUGAUUUCUAAUUAUACAAAAUGAACUAUA
AGUGGUCUGUUUUGCAAGGAAACAGGAUUAUUUCUAAUUUACUGGCAUCAAAGAAGUUCAUGGAGUGCCAG
4
UUCCCCACAUGUGCUAAUAAAAGUCGCCUUUUUAUUUUGAAACAGUGUGCCAAAAGAGGGUUAGCAUUUCCU
GACCUGCGUUUUUGGCUGUCGUACAACCCAAAGUAACUCGAUUUGGCUUUUUUUUUAGUUUUUUAUUUUAC
CGAAAACAUCUACAUCUGGUUUCAGAACACUUUUAAAUUUACUGGAAUUGUUAAAUUCAAGAAGUUAACUACA
AGAGAGAAUAAUUACAUUGACAAGUAUGAGCUGGCAGCCUUUGUUUCAGUGGUUAUUUUCUGAAUACAACUU
UAAUGACAAGUGGUCUUUAGUGGUUGGGACAAAAUGUAUUAGUCAGCUGCAGAAACAGAAGUGCCGUCACAA
AGAACAGACACAUAUGGAUACCUACAACUAUCAUGCAAAAAGUAACGCACUUUAUGUCCCAUGUUUUAACGCA
5 6
AAAAAUCUAUUUGGUAGCUCUGUUUGUGUUUUGUGUCUCACUUUUAUUUUCUUUUCUAAGCUUUUCUCAAG
GUUCCUGUUGCAUUUGUAGCCGGCAAAAAUCUGAGACAGCUAUACAUCUAUUGUUCAAAUUGUCAAAAUCUA
7
AAUUGAAUGUUAUGCAAAUGUUUUAUUUUUGUACUCCUAAGCAUUUAUAUAUUGGGUGCAAACAUUUAUAUG
AAGGAACUGUAUACUUUCACAACUGAGCCAUCCUGCUUUUUUUUGUAAACCUGUAUCACAGCUGCUGAGUAA
8 9
AAACAAGACAUUUUUAAAAGUCA

B

Region1

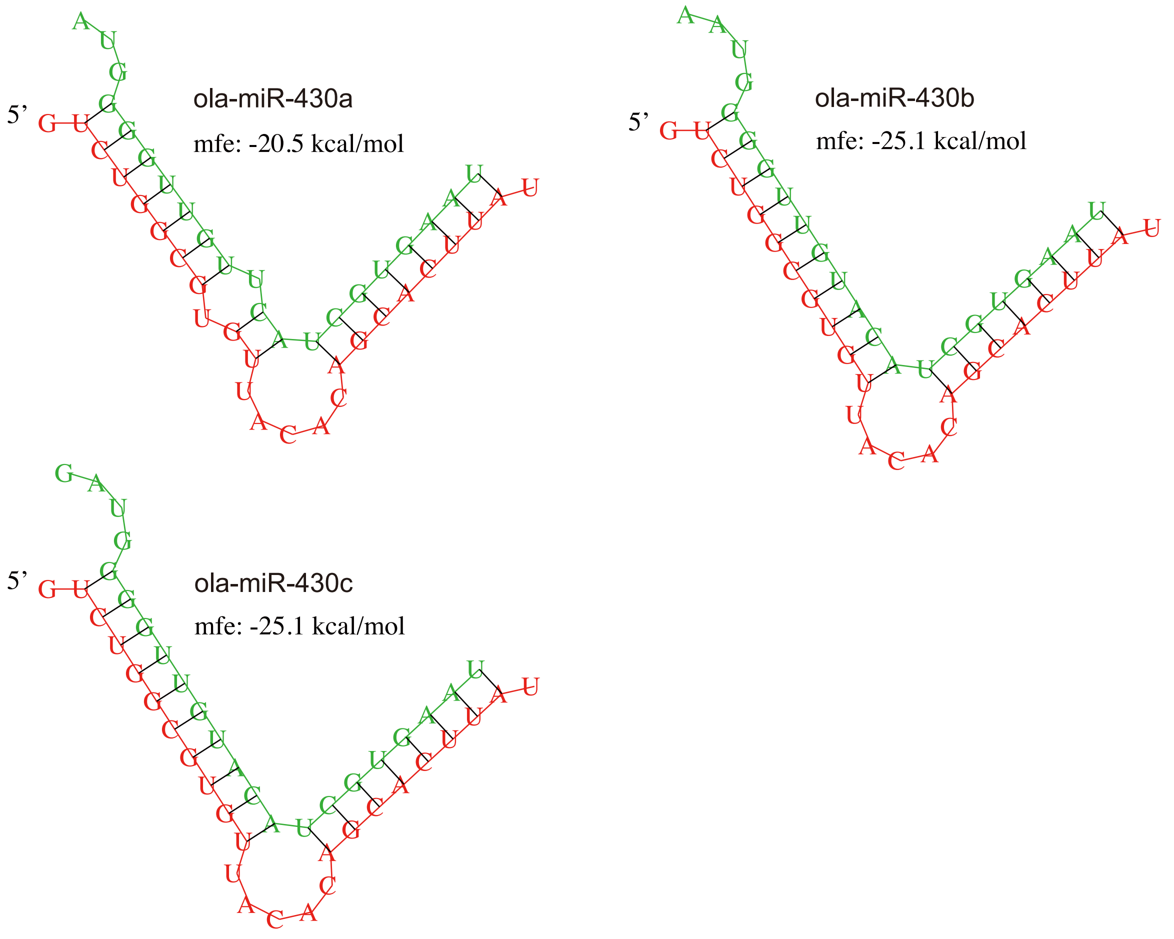

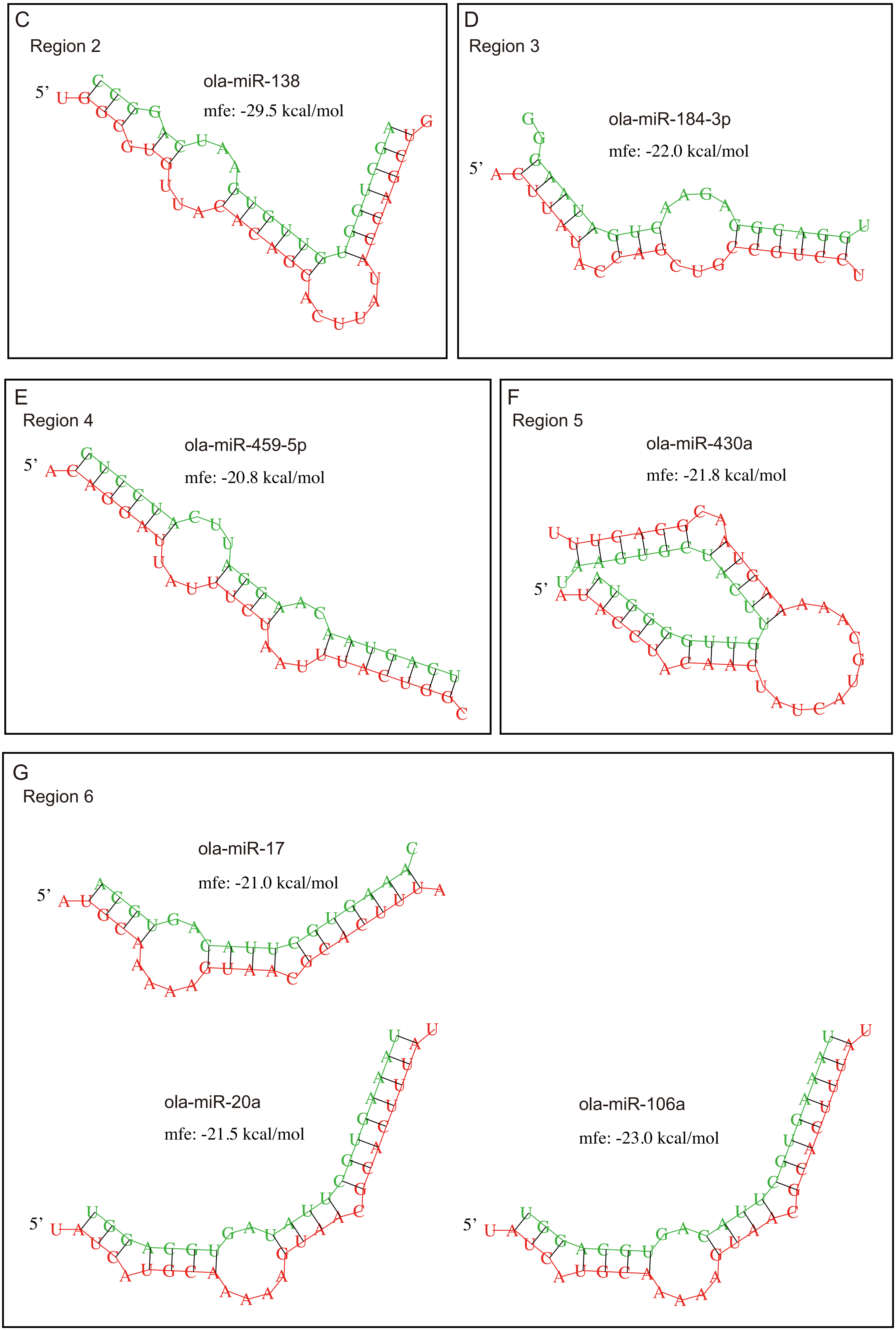

C
Region 2
ola-miR-138
mfe: -29.5 kcal/mol
D
Region 3
ola-miR-184-3p
mfe: -22.0 kcal/mol
E
Region 4
ola-miR-459-5p
mfe: -20.8 kcal/mol
F
Region 5
ola-miR-430a
mfe: -21.8 kcal/mol
G
Region 6
ola-miR-17
mfe: -21.0 kcal/mol
ola-miR-20a
mfe: -21.5 kcal/mol
ola-miR-106a
mfe: -23.0 kcal/mol

H

Region 7

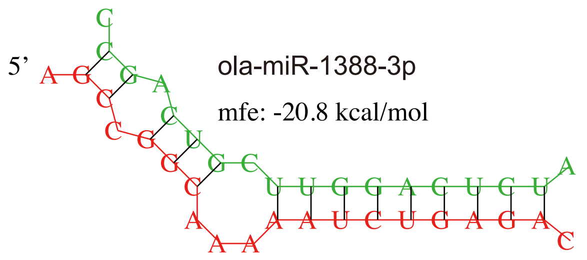

I

Region 8

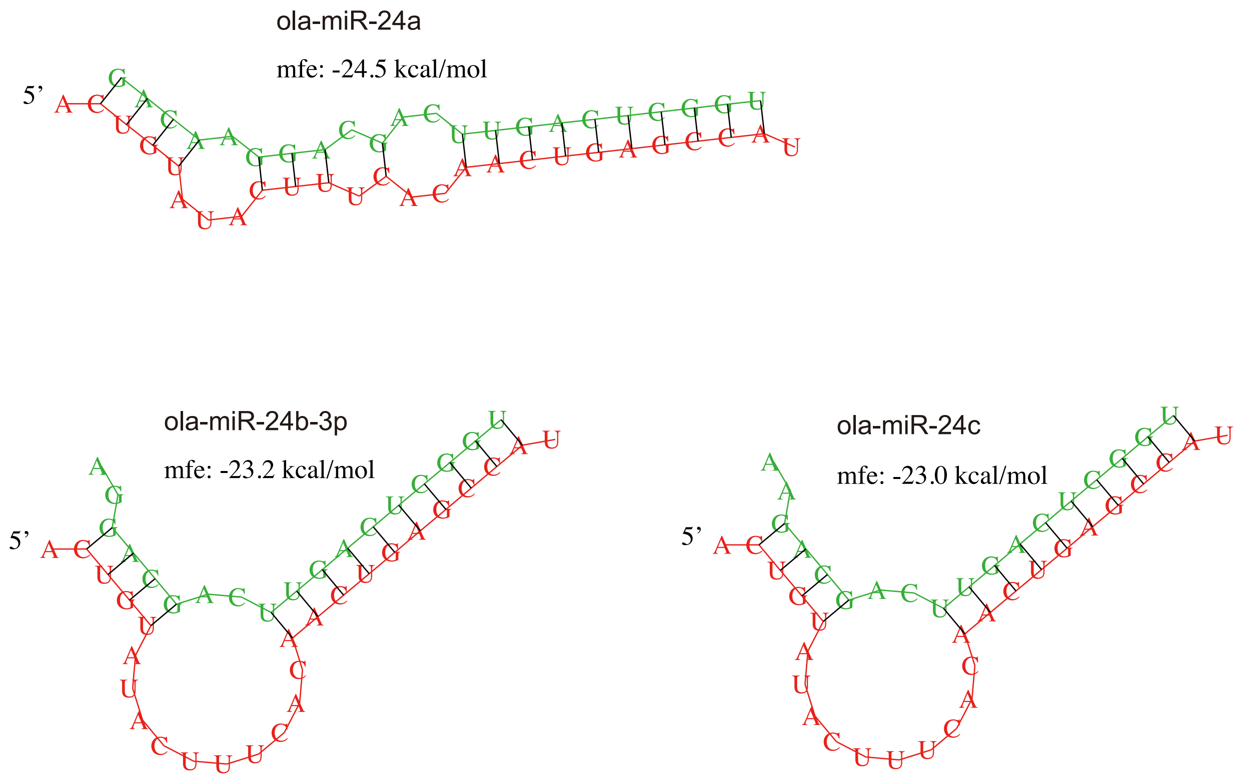

J

Region 9

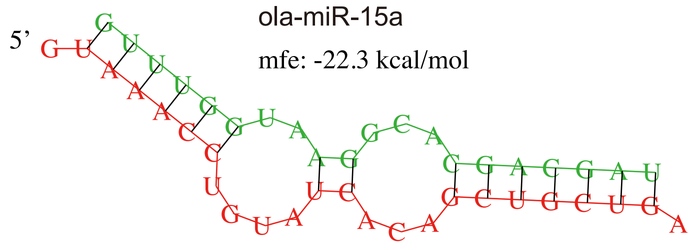

Supplement: S2 Fig — A. A 1,317b RNA sequence in the sdf1a 3' UTR. The red triangle shows the end of the 371b length of the short 3' UTR found in the nar mutant embryos. Red letters and underlined regions 1–9 show predicted targets for the seed regions of the miRNAs registered in the miRNA database. B-J. Predicted secondary structures of the miRNA/target duplexes in the target regions 1–9 shown in A. the green sequences are miRNAs and the red sequences are the target 3'UTRs. Minimum free-energy (mfe) values of the hybridizations and the names of the miRNAs are shown in the figures. (PDF) [file pone.0172467.s002.pdf]
