# Supplementary material for: Mutation in cpsf6/CFIm68 (Cleavage and Polyadenylation Specificity Factor Subunit 6) causes short 3'UTRs and disturbs gene expression in developing embryos, as revealed by an analysis of primordial germ cell migration using the medaka mutant naruto
Source: PLoS One. 2017 Mar 2;12(3):e0172467. doi: 10.1371/journal.pone.0172467 (PMC5333813; doi:10.1371/journal.pone.0172467)
Supplement: S1 Table — Primer information used in the genetic linkage mapping of the narj113-2B mutation. (DOCX) [file pone.0172467.s004.docx]

**S1 Table. Primer information used in the genetic linkage mapping.**

| Marker name | 5' primer sequence | 3' primer sequence | Used restriction enzyme* | Recombination frequency** |
| --- | --- | --- | --- | --- |
| NAV3_F01R01 | ACAGATTGGGCCAACCACTA | ATACATGGAAAACGCGCAAA | DdeI | 13/192 |
| AU172060 | TTTTTGGATCTTTTGTTGGGGTCCG | CCAAAGGCTTTAAGGTTCTTCCTCCCT | DdeI | 5/190 |
| PHTF2_F01R01 | TGTCGCATGGAAAATGAAAG | ATCTGCTATGCTGCTGCTGA | indel | 2/192 |
| ERC1b_F01R01 | CCCACAACTCAGAGGCGTAT | TGTTTTGCAGTATGGGTGGA | indel | 5/1716 |
| WNK1a_F01R01 | GTCCTCTACAATCCCCCACA | TGCAACTGAAACCAAAAACAA | indel | 4/1716 |
| chr23-14.19Mb_F01R01 | GGCTTGAGACTCCAAACAGG | GGACTTGTAGATGTGTGAATGTGG | indel | 3/1716 |
| NAPEPLD_F01R01 | TGACCTGTTGGCATTTGTGT | CTCCATGAGTCTCCCACCTC | DraI | 2/1716 |
| CPSF6 3'UTR_F01R01 | CGGTGCCTCATTTTCTTACC | TGCTTAAGGTCCCATGTGAAG | indel | 1/1716 |
| CPSF6_F01R01 | TGCGTGGATGTTGTTTGTGT | ACCAGCACAAGACACCCTGT | indel | 0/1716 |
| CACNA2D4b_F01R01 | TTGTGTGGCGAATTCAAGAG | GCGAGATTTGCACCACTTTT | indel | 1/1716 |
| LRTM2_F01R01 | GGATAATTTGCCTGGTGGATT | TCATCAAGGAGACCCAGAGG | indel | 1/1716 |
| DCP1B_F01R01 | ATCGTATGTGGACGCAAGGT | TGTGGGAGAATATGGGCTGT | indel | 2/1716 |
| CACNA1C_F01R01 | CGTCCACTCTCTGACATCCA | TCGTTCTTGACTGGGTCCTC | TaqI | 7/1716 |
| TSPAN33_F01R01 | GCTGGTATCATGGGCTTCAT | TGGCCATAATCGATGAGGTT | indel | 7/1716 |
| SMO_F01R01 | CACAAGGACCCAGAGAAGGA | CGGCTCGTTTATGTCGAAGT | indel | 9/1716 |
| PLXNA4_F02R02 | ATTGAGCCAGAGTGGAGCAT | ATTGGTTCCTGTCACCGTCA | DraI | 6/954 |

*Used restriction enzyme of the PCR-RFLP (restriction fragment length polymorphism) markers. "indel" means insertion-deletion markers.

**Recombination frequency between the *nar^j113-2B^* and the markers.
